# A two-dimensional grating surface made of quaternary Huygens elements excited by a real source

**DOI:** 10.1038/s41598-023-34453-9

**Published:** 2023-05-08

**Authors:** Iman Derafshi, Nader Komjani, Ensiyeh Ghasemi Mizuji

**Affiliations:** 1 Department of Electrical Engineering, Iran University Science and Technology, 16846-13114 Tehran, Iran; 2 Department of Electrical and Computer Engineering, Tarbiat Modares University, 14115-111 Tehran, Iran

**Keywords:** Electrical and electronic engineering, Applied physics

## Abstract

In this article, a new method to create an anomalous reflection in the desired direction is proposed. Two-dimensional grating surfaces consisting of four particles with the properties of a Huygens source are employed in each period. Then, this method is extended to the problem in which the grating surface is illuminated by a real source such as a horn. In this case, the designed grating surface has different periods in both directions to collimate the reflected wave and give an in-phase wavefront. Using our method, a high-efficiency reflectarray (RA) based on quaternary Huygens grating is designed. This RA is distinguished from common RAs due to its beam squint capability. This array offers more aperture efficiency and thus more gain in comparison to the leaky waves that inherently have low aperture efficiency. Therefore, our designed RA can compete with leaky wave antennas in many applications. The mentioned RA is designed to have the main beam in the direction of $\left( {{\theta _0} = {{68}^{\circ }},{\varphi _0} = {{225}^{\circ }}} \right)$, at the frequency of 12 GHz. The simulation results show the realized gain and SLL of this antenna are 24.8 dB and $-15.5$ dB, respectively. Also, by changing the frequency in the range of 12–15 GHz, the direction of the main beam varies from $\left( {{{68}^{\circ }}, {{225}^{\circ }}} \right)$ to $\left( {{{39}^{\circ }}, {{225}^{\circ }}} \right)$.

## Introduction

Metasurfaces, the periodic structures made of sub-wavelength elements, have attracted many researchers’ attention, in recent years^1–3^. Recently, Huygens metasurfaces (HMS) are introduced in which the constituent particles are Huygens sources. Huygens sources are a superimposing of subwavelength electric and magnetic dipoles and create unidirectional radiation^4,5^. Using these Huygens sources in designing meta-surface results in improvement of the transmission efficiency in the transmission mode^6^. Many articles study HMSs excited by the plane waves, in transmission mode^7–9^. However, in many realistic problems, the meta-surfaces are not illuminated by a plane wave. In Ref.^10^, HMSs excited by real sources are investigated. These metasurfaces are applicable in many applications; such as transmit-array antennas^11,12^, leaky wave antennas^13^, and other applications^14^. On the other hand, it is a little more difficult to design Huygens metasurfaces in reflective mode. Since, for a surface to have a perfect anomalous reflection, the normal component of the Poynting vector must follow the sinusoidal function^15^. Therefore, the surface absorbs power in some areas which means that the vertical component of the Poynting vector is negative, and the power should be irradiated in other areas. To design such a surface using passive Huygens metasurfaces, a method based on employing auxiliary evanescent waves is presented in Ref.^16^. However, as it is mentioned in Ref.^17^, the explained design has a complex concept and hard implementation since the resulting metasurface requires deep subwavelength element dimensions and more than one layer. In Ref.^17^, it is tried to present a simple structure with easier implementation using Binary Huygens Metasurfaces (BHMs). In that article, using power flow analysis, it is shown that the designed BHM meets the necessary condition to have a perfect anomalous reflection. The introduced BHMs up to now^17,18^ are one-dimensional and designed to work under the illumination of plane wave incidence. However, the problem of developing these meta-surfaces into 2D structures under the illumination of real excitations steel remained unsolved; therefore, it is difficult to design them in applications such as Reflectarrays.

RAs are preferred to parabolic reflector antennas in many applications due to their flat surface, low weight, and low manufacturing cost^19–21^. The main drawbacks of these antennas are their low bandwidth and efficiency. In order to solve these problems, many efforts have been done in recent years^22,23^. Common RAs are usually composed of an array of unit cells with a constant array period, and each of these unit cells has one or more resonant elements^24^. The required 360-degree phase variation for designing an RA is created by adjusting one or more parameters in its constituent resonant elements. Various methods are used for this purpose, such as: changing the dimensions of the elements^25^, elements loaded by delay lines^26^, element rotation^27^, aperture coupled patch^28^, and Phoenix elements^29^. Moreover, RAs are applicable in different functions such as polarization control^30^, creating multiple beams^31^, dual or multi-band structures^32^, beam shaping^33^, and other applications^34,35^. Another application of RAs is beam scanning. Although many efforts are done for mechanical and electrical beam scanning in RAs^36–38^, the possibility of beam squint has rarely been investigated in these arrays. In Ref.^39^, it is shown that to achieve the beam squint capability in RAs, it is necessary to change the feed position mechanically. Recently, Refs.^40–42^ present an interesting method based on providing a unit cell with the ability to control the slope of the $S_{11}$ phase curve along with providing the range of phase variation required to control the main beam direction of the antenna and have frequency scanning ability. Although these articles provide the advantage of beam frequency scanning control, they suffer from the complexity in designing the unit cells; and as it is mentioned in those papers, there are actually significant limitations on the maximum achievable sizes in the x- and y-axises directions.

In this paper, in the first step, the design procedure of 2D grating surfaces excited by a plane incident wave is developed. These surfaces are included in the quaternary Huygens sources in each period. The structures designed using this method are known as quaternary Huygens grating surfaces (QHGS) throughout this article. In the next step, this design method is extended to the realistic applications illuminated by a real source such as a horn. Using this generalized method, a high-efficient RA can be designed in near-grazing reflection angles. In this article without losing generality, an RA is designed to have the main beam at ($\theta _o=70^\circ$, $\phi _o=225^\circ$). In this RA, the unit cell periods have variable values and there is no need for resonant elements to create the required 360-degree phase variation. In our design, simple elements such as single-layer dipoles are employed. These elements should have the Huygens property as it is mentioned in Ref.^43^. In addition, the RA designed by this method has the beam squint capability and in comparison to Ref.^39^, there is no need to change the feed position for beam squint. Moreover, in comparison to Ref.^40^, our designed unit cell is simpler and there is no limitation on the antenna dimension. We believe this work is a step forward in designing high-efficient RAs with beam squint capability. Our designed antenna has great application in modern telecommunication systems, mobile systems, radar systems, and satellite systems in which beam scanning capability is required^44^. In addition, with an appropriate unit cell, this method can be used to achieve polarization conversion capability.

## Process of designing an QHGS

**Figure 1:**
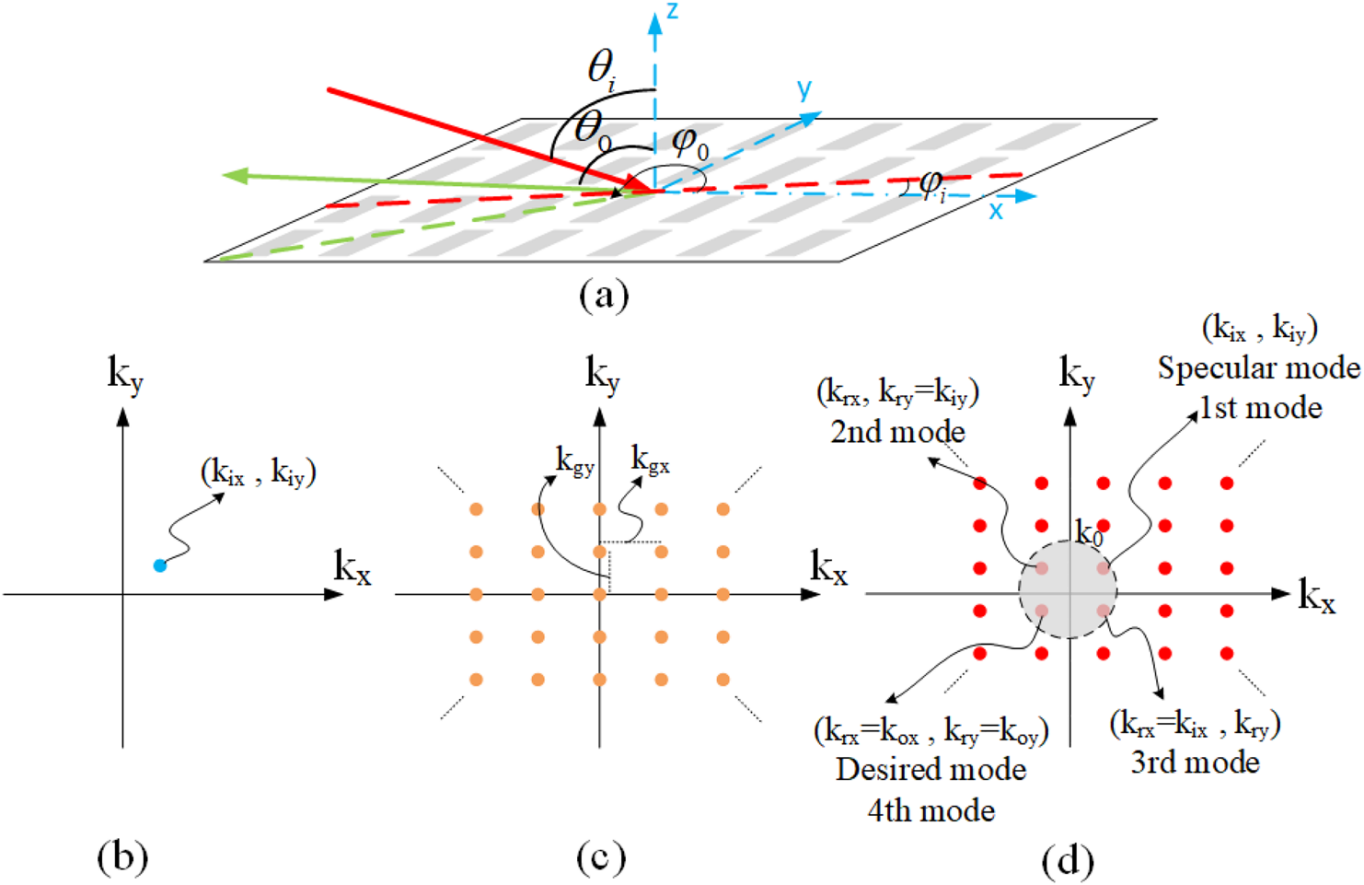
(**a**) Schematic of the overall structure. Describing diagram of the *k*-domain for (**b**) radiating plane wave, (**c**) grating surface with the rectangular arrangement, and (**d**) reflected waves of grating surface which illuminated by a plane wave incidence.

In order to create an anomalous reflection, in this section, our proposed method to design a QHGS under the illumination of a plane wave is introduced. For a periodic structure with a rectangular arrangement (Fig. 1a), the expansion of the Floquet series in the *k*-domain is as follows^45^:

1 $$\begin{aligned} \tilde{h}({k_x},{k_y}) = \frac{{4{\pi ^2}}}{{{\Lambda _{gx}}{\Lambda _{gy}}}}\sum \limits _m {\sum \limits _n {\delta \left( {{k_x} - \frac{{2\pi m}}{{{\Lambda _{gx}}}}} \right) } } \delta \left( {{k_y} - \frac{{2\pi n}}{{{\Lambda _{gy}}}}} \right) \end{aligned}$$

where ${\Lambda _{gx}}$ and ${\Lambda _{gy}}$ are the periods of the structure in the x and y directions, respectively. In Fig. 1c, the diagram in the *k*-domain is displayed, completely; where ${k_{gx}}$ and ${k_{gy}}$ are equal to $2\pi \big /\Lambda _{gx}$ and $2\pi \big /\Lambda _{gy}$, respectively. In Fig. 1d, the *k*-domain diagram of the reflected waves is shown for the structure under the illumination of TE plane wave incidence in Fig. 1b. As it is seen in this figure, only the modes inside the propagation region (within the circle with a radius equal to ${k_0}$) are propagating and other modes are evanescent. The design of a QHGS has two stages. The first stage is to adjust the dimensions of the periods and the incident angle to have the minimum number of propagating modes that are included in the desired mode. In the second stage, the elements inside each period are appropriately designed to eliminate undesirable propagating modes; therefore, only one propagating mode remains.

### Determining the periods and the incident angle

In the proposed method, the minimum number of propagation modes is achieved by modifying the structure periods in the *x* and *y* directions. By knowing that the specular reflection mode (1st mode) is always propagated, to have the minimum number of propagating modes, one of the three closest modes to the specular reflection mode is considered as the desired mode. These three closest modes to the specular reflection mode are specified in Fig. 1d. To have a propagating mode in the desired direction, its wave number should be independent of the wave number of the incident wave. In the 2nd/3rd mode, $k_x$/$k_y$ is independent of $k_x$/$k_y$ of the incident wave but its $k_y$/$k_x$ is equal to that of the incident wave. However, for the 4th mode, both $k_x$ and $k_y$ can be adjusted independently of the *k* components of the incident wave. Therefore, the 4*th* mode is a suitable choice to be considered as the desired mode. In this case, the necessary conditions to have the 4*th* mode in the desired direction $\left( {{\theta _0},{\varphi _0}} \right)$ are as follows^45^:

2 $$\begin{aligned} {k_{rx}}= & {} {k_{ox}} = {k_0}\sin \left( {{\theta _o}} \right) \cos \left( {{\varphi _o}} \right) \nonumber \\ {k_{ry}}= & {} {k_{oy}} = {k_0}\sin \left( {{\theta _o}} \right) \sin \left( {{\varphi _o}} \right) \end{aligned}$$

Therefore, according to Fig. 1d, ${k _{gx}}$ and ${k _{gy}}$ are calculated using the following equation:

3 $$\begin{aligned} {k_{gx}}= & {} \left| {{k_{ox}} - {k_{ix}}} \right| = {k_0}\left| {\sin \left( {{\theta _o}} \right) \cos \left( {{\varphi _o}} \right) - \sin \left( {{\theta _i}} \right) \cos \left( {{\varphi _i}} \right) } \right| \nonumber \\ {k_{gy}}= & {} \left| {{k_{oy}} - {k_{iy}}} \right| = {k_0}\left| {\sin \left( {{\theta _o}} \right) \sin \left( {{\varphi _o}} \right) - \sin \left( {{\theta _i}} \right) \sin \left( {{\varphi _i}} \right) } \right| \end{aligned}$$

As a result, according to $k_{gx}=2\pi \big /\Lambda _{gx}$ and $k_{gy}=2\pi \big /\Lambda _{gx}$, the periods of the structure in both the *x* and *y* directions are calculated as follows:

4 $$\begin{aligned} {\Lambda _{gx}}= & {} {{{\lambda _0}}\big /\left| {\sin \left( {{\theta _o}} \right) \cos \left( {{\varphi _o}} \right) - \sin \left( {{\theta _i}} \right) \cos \left( {{\varphi _i}} \right) } \right| }\nonumber \\ {\Lambda _{gy}}= & {} {{{\lambda _0}}\big / \left| {\sin \left( {{\theta _o}} \right) \sin \left( {{\varphi _o}} \right) - \sin \left( {{\theta _i}} \right) \sin \left( {{\varphi _i}} \right) } \right| } \end{aligned}$$

In the next step, to determine the suitable incident angle, the number of modes inside the propagation region is checked for different values of $\left( {{\theta _i},{\varphi _i}} \right)$. The number of described modes would be less than the value of $N_t$ obtained from the following equation:

5 $$\begin{aligned} {M_x} = \left\lfloor {\frac{{2{k_0}}}{{{k_{gx}}}}} \right\rfloor \,\,\,,\,\,\,{N_y} = \left\lfloor {\frac{{2{k_0}}}{{{k_{gy}}}}} \right\rfloor \,,\,\,\,\,{N_t} = {M_x} \times {N_y} \end{aligned}$$

This equation determines the number of modes inside a square of $2k_0\times 2k_0$, with the center located at the origin of the coordinates of Fig. 1d. Therefore, the modes located in the propagating region (circle with radius $k_0$) are also located inside this square. As a result, to determine the number of propagating modes more accurately, the condition of Eq. (6) should be checked. For each *mnth* mode, if this condition is met, it is propagating, otherwise, it is evanescent.

6 $$\begin{aligned} \sqrt{{{\left( {{k_{ix}} + m{k_{gx}}} \right) }^2} + {{\left( {{k_{iy}} + n{k_{gy}}} \right) }^2}}< {k_0}\,\,\,\,\,\,\,\,\,\,\, - {M_x}< m< {M_x}\,,\,\,\, - {N_y}< n < {N_y} \end{aligned}$$

**Figure 2:**
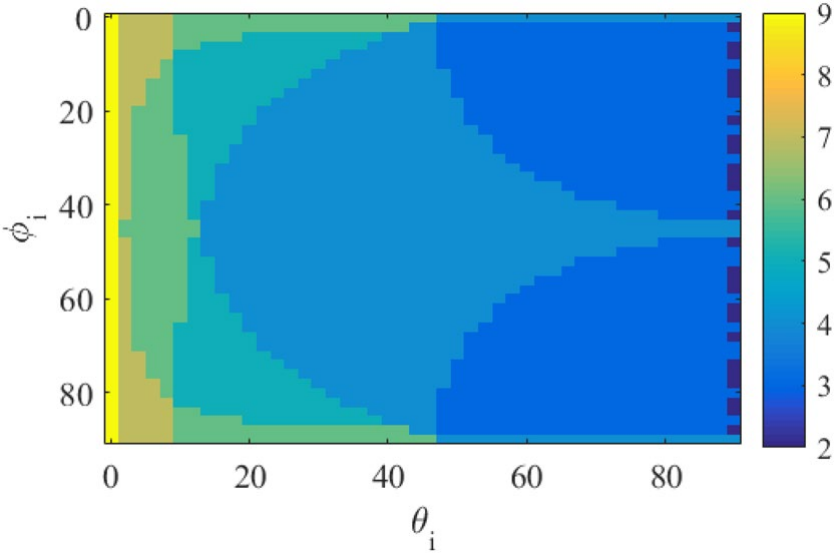
The number of propagation modes versus different incident angles to have the desired mode in direction of $\left( {{\theta _0} = {{70}^{\circ }},{\varphi _0} = {{225}^{\circ }}} \right)$.

**Figure 3:**
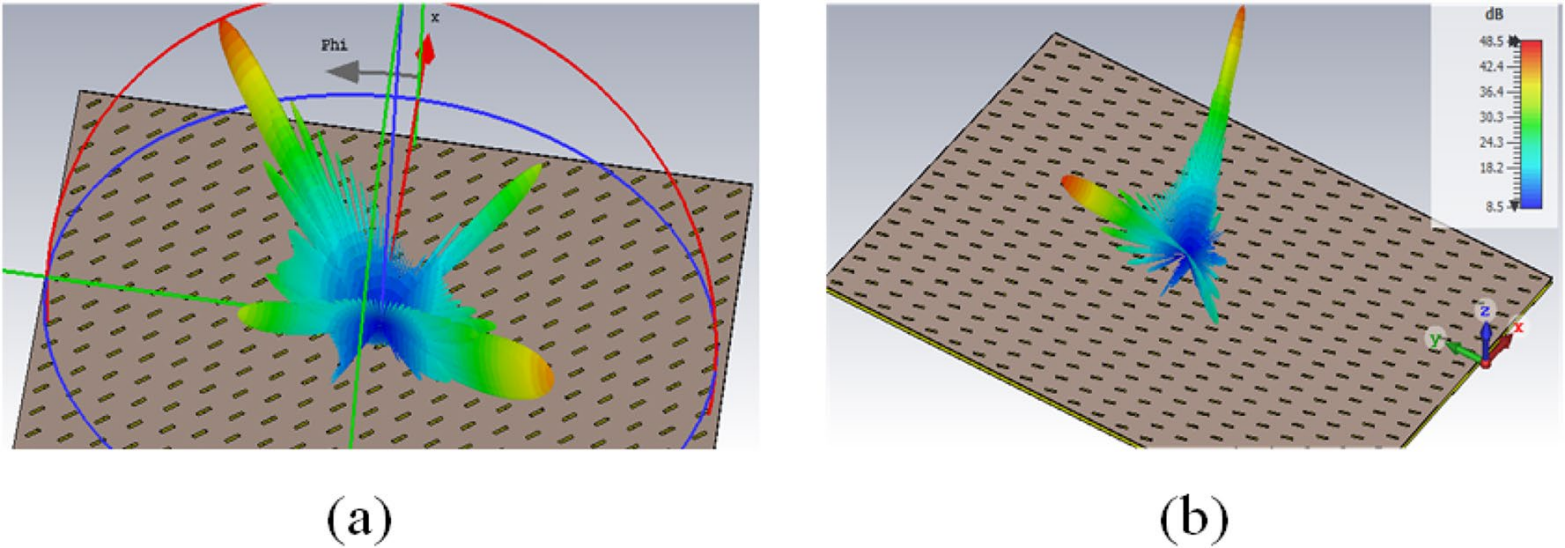
(**a**) Four propagated modes for the incident wave in the direction of $\left( {{\theta _i} = {{61.5}^{\circ }},{\varphi _i} = {{45}^{\circ }}} \right)$ and (**b**) three propagated modes for the incidence in the direction of $\left( {{\theta _i} = {{51}^{\circ }},{\varphi _i} = {{16}^{\circ }}} \right)$.

To have the reflected wave in the desired direction of $\left( {{\theta _0} = {{70}^{\circ }},{\varphi _0} = {{225}^{\circ }}} \right)$, the number of propagation modes for different values of incident angle of $\left( {{\theta _i},{\varphi _i}} \right)$ is plotted in Fig. 2 using Eqs. (5) and (6). As can be seen in this figure, for most values of $\left( {{\theta _i},{\varphi _i}} \right)$, there are three or four propagating modes (e.g, the number of propagating modes is equal to four for $\left( {{\theta _i} = {{25}^{\circ }},{\varphi _i} = {{45}^{\circ }}} \right)$. To demonstrate this issue, two arrays have been designed and simulated to reflect the wave in the desired direction of $\left( {{\theta _0} = {{70}^{\circ }},{\varphi _0} = {{225}^{\circ }}} \right)$. The first and second arrays are stimulated under the illumination of a plane wave with an incident angle of $\left( {{\theta _i} = {{61.5}^{\circ }},{\varphi _i} = {{45}^{\circ }}} \right)$ and $\left( {{\theta _i} = {{51}^{\circ }},{\varphi _i} = {{16}^{\circ }}} \right)$, respectively. In these arrays, it is assumed that a printed dipole on a grounded dielectric with the same dimensions in each period is employed as a unit cell. As is seen in Fig. 3, the number of propagating modes, for the first and second arrays are four and three, respectively. In the first array, these four modes are the specular reflection mode (with ${{k_x} = {k_{ix}},{k_y} = {k_{iy}}}$), the desired mode (with ${{k_x} = {k_{ox}},{k_y} = {k_{oy}}}$), and the other modes with $\left( {{k_x} = {k_{ox}},{k_y} = {k_{iy}}} \right)$ and $\left( {{k_x} = {k_{ix}},{k_y} = {k_{oy}}} \right)$, respectively. Using Eq. (7), the propagation directions of these modes can be easily calculated which are $\left( {{{61.5}^{\circ }},{{45}^{\circ }}} \right)$, $\left( {{{70}^{\circ }},{{225}^{\circ }}} \right)$, $\left( {{{65.5}^{\circ }},{{137}^{\circ }}} \right)$, and $\left( {{{65.5}^{\circ }},{{313}^{\circ }}} \right)$, respectively. For the second array, three propagation modes are the specular reflection mode, the desired mode, and an unwanted mode with $\left( {{k_x} = {k_{ox}},{k_y} = {k_{iy}}} \right)$. The last mode propagation angle is $\left( {{{44}^{\circ }},{{162}^{\circ }}} \right)$, using Eq. (7). To control these three or four modes and remove unwanted modes, 3 or 4 degrees of freedom are required in the design of the unit cell, respectively. This issue is discussed in the next subsection.

7 $$\begin{aligned} {\theta _{eq}} = {\sin ^{ - 1}}\left( {\sqrt{\frac{{k_x^2 + k_y^2}}{{k_0^2}}} } \right) ,\,\,\,\,\,\,\,\,\,{\varphi _{eq}} = {\tan ^{ - 1}}\left( {\frac{{{k_y}}}{{{k_x}}}} \right) \end{aligned}$$

### Unit cell design

**Figure 4:**
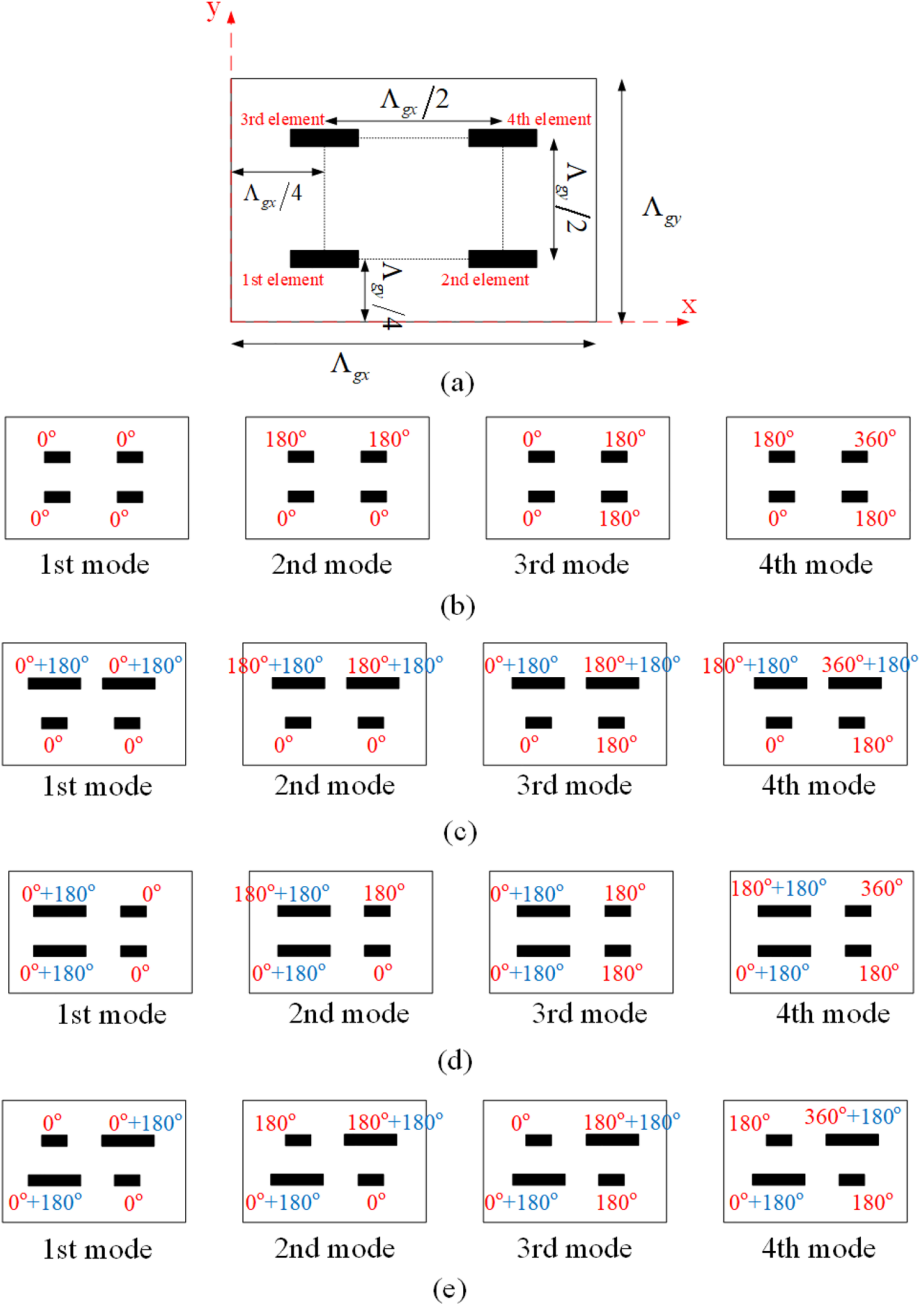
(**a**) Placement of elements in a period. The phase error due to the different path lengths for each element in a period for the reflective mode (**b**) first, (**c**) second, (**d**) third, and (**e**) desired and for radiation angle of $\left( {{\theta _i} = {{61.5}^{\circ }},{\varphi _i} = {{45}^{\circ }}} \right)$ and optimum reflection angle of $\left( {{\theta _0} = {{70}^{\circ }},{\varphi _0} = {{225}^{\circ }}} \right)$.

To control each mode as indicated in Ref.^17^, it is required to have at least one degree of freedom in the unit cell design. For example, four degrees of freedom are essential for the first array simulated in the previous subsection; since in that array, there were four propagating modes in directions of $\left( {{{61.5}^{\circ }},{{45}^{\circ }}} \right)$, $\left( {{{70}^{\circ }},{{225}^{\circ }}} \right)$, $\left( {{{65.5}^{\circ }},{{137}^{\circ }}} \right)$, and $\left( {{{65.5}^{\circ }},{{313}^{\circ }}} \right)$ (as shown in Fig. 3a). With this in mind, in this article, four dipoles with variable lengths are used in a unit cell. The location of these four dipoles in the unit cell is specified in Fig. 4a.

It is assumed to have the incident and reflected waves in the directions of $(\theta _i,\varphi _i)$ and $(\theta _r,\varphi _r)$, respectively. The phase difference due to the path length difference between any two elements which are placed in different positions with a spatial difference of $\left( {\Delta x,\Delta y} \right)$ can be easily obtained using the ray tracing method and as follows:

8 $$\begin{aligned} \Delta \theta = k\left( {\Delta x\left( {\sin {\theta _i}\cos {\varphi _i} - \sin {\theta _r}\cos {\varphi _r}} \right) + \Delta y\left( {\sin {\theta _i}\sin {\varphi _i} - \sin {\theta _r}\sin {\varphi _r}} \right) } \right) \end{aligned}$$

here, the first element in Fig. 4a is chosen as a reference and the phase differences are calculated in comparison to this element using Eq. (8). These phase differences for all of the four modes are calculated as shown in Fig. 4b. For the first mode (specular reflection), the total phase difference between the constituent elements in a unit cell is zero; therefore, this mode is propagated. For the 2nd and 3rd modes (undesirable modes), as is seen in this figure, the elements placed on each diameter are in opposite phases. So, two elements are in phase, and the other two elements have $180{^{\circ }}$ of phase differences from them. In this situation, if the elements are the same, the reflected waves from these elements cancel each other. Therefore, these two modes are not propagated. For the fourth mode (the desired mode), the situation is similar to the previous two modes. In this case, the elements placed in each diameter are in phase, and there is a phase difference of $180^\circ$ between the elements placed in different diameters. Consequently, if our unit cell is consisting of similar elements, the desired mode is not propagated. Therefore, under these conditions, only the specular reflection mode is propagated.

The phase differences between the elements can be changed, by adjusting the length of the dipoles. Therefore, it is possible to adjust the phase difference between elements to suppress the specular reflection mode and make each of the three other modes propagate. For this purpose, it is necessary to change the length of two of the dipoles to create $180{^{\circ }}$ of phase difference in comparison to the other two dipoles. By proper choice of these two dipoles, the propagating mode is specified. In Fig. 4c–e, the created phase differences for three different choices are shown. In the first case (Fig. 4c), it is assumed that the dimensions of the third and fourth dipoles are adjusted to create a 180-degree phase difference in comparison to 1st and 2nd elements. In this case, the total phase difference for different modes obtained by adding these phase differences to the path phase differences is shown in Fig. 4c. As it is clear from this figure, for the second mode, all elements are in phase and this mode is propagated; while for the other modes, two elements are in phase and the other two elements have opposite phases. Therefore, the other modes are not propagated. The same explanation can be brought for the phase differences shown in Fig. 4d or e. From these figures, if 2nd and 4th elements or 2nd and 3rd elements create 180-degree phase differences in comparison to the remaining two other elements, only the third or fourth mode would be propagated, respectively. Since the fourth mode is our desired mode, it is necessary to adjust the dimensions of the 2nd and 3rd elements to create 180-degree phase differences in comparison to the 1st and 4th elements as shown in Fig. 4e.

**Figure 5:**
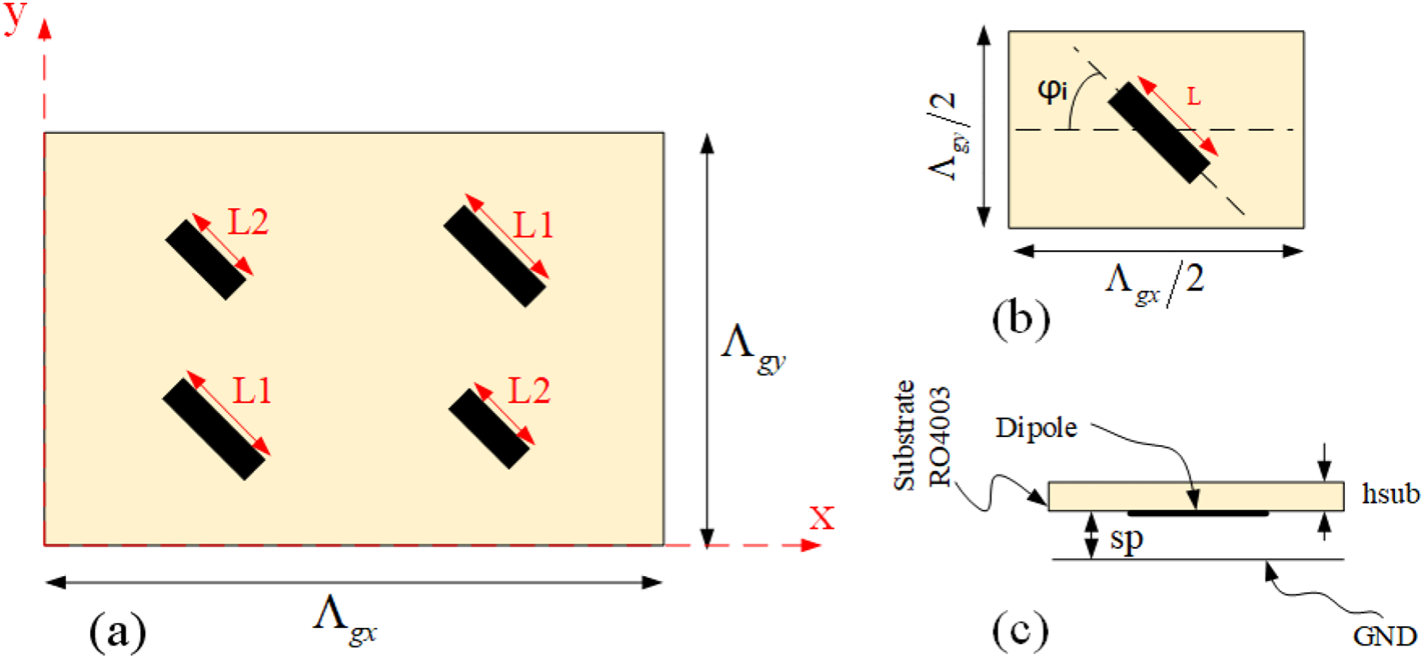
(**a**) Arrangement of elements in a period to have the desired propagating mode. (**b**) Top view and (**c**) side view of the sub-unit cell which is used to obtain the dimensions of the dipoles.

**Figure 6:**
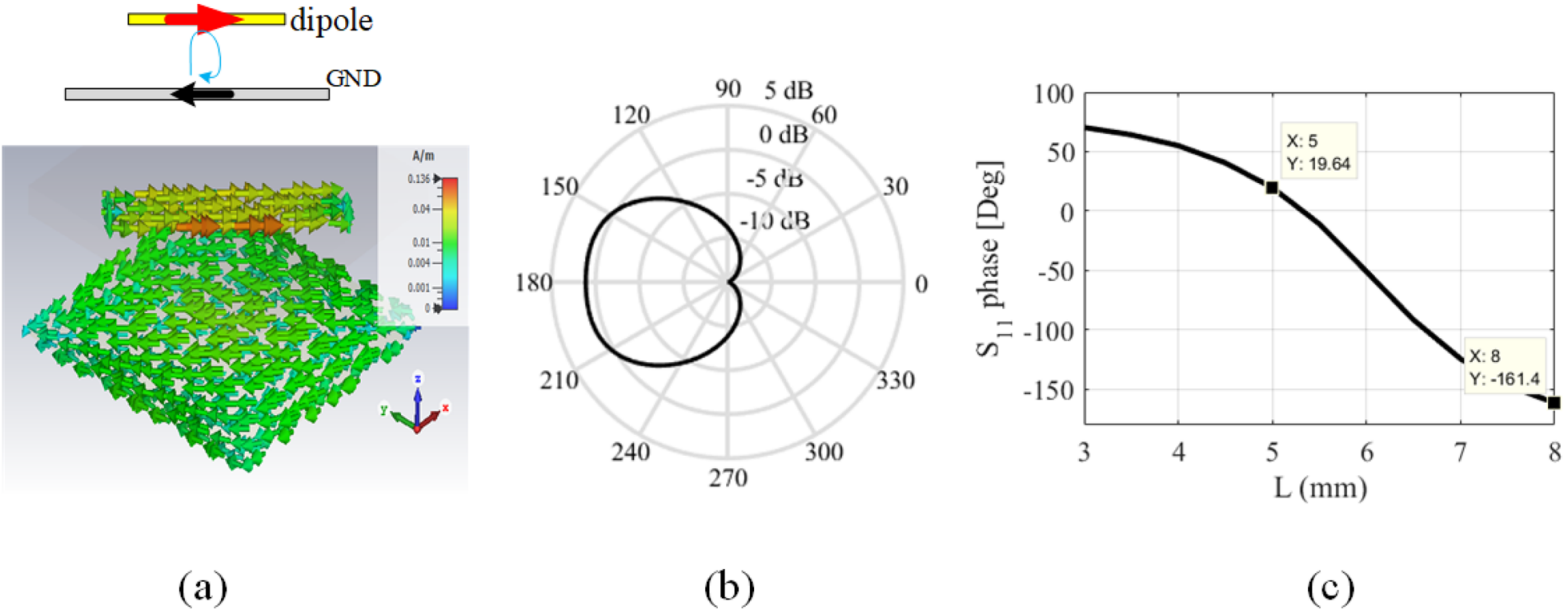
(**a**) Current distribution of the sub-unit cell, (**b**) Unidirectional pattern of the isolated sub-unit cell, and (**c**) variation of the reflected wave phase versus dipole length with the assumption of an infinite array.

**Figure 7:**
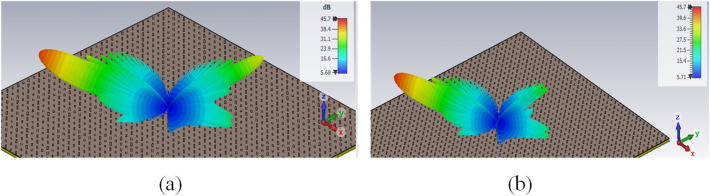
Array pattern (**a**) before and (**b**) after optimization of the L2 parameter, under the illumination of a plane wave with an incident angle of $\left( {{\theta _i} = {{61.5}^{\circ }},{\varphi _i} = {{45}^{\circ }}} \right)$ and desired reflection angle of $\left( {{\theta _0} = {{70}^{\circ }},{\varphi _0} = {{225}^{\circ }}} \right)$.

According to the mentioned points, the final arrangement of the elements inside each unit cell is shown in Fig. 5. The length of the 1st and 4th dipoles are considered equal to *L*1, and the lengths of the 2nd and 3rd dipoles are equal to *L*2, and these dipoles are oriented parallel to the incident electric field. The length difference between *L*1 and *L*2 is adjusted to create a 180-degree difference between these elements. To adjust the length of the dipoles, a sub-unit cell consisting of only one dipole is considered as shown in Fig. 5b,c. A RO4003 ($\varepsilon _r=3.55, \tan \delta =0.0027$) with 32 mil thickness is employed as a substrate. The substrate is placed at a specific air distance of *sp* from the ground plane and the dipole is placed between this air distance and the substrate (as shown in Fig. 5c). Here, similar to Ref.^43^, Huygens elements are used to design highly efficient grating surfaces. A necessary condition for an element to be a Huygens source is that its isolated sub-unit cell has a unidirectional radiation pattern. To reach this purpose, three parameters of *L*, *w*, and *sp* are optimized. The optimal values of *L*, *w*, and *sp* are obtained using CST software as 3.5 mm, 1.5 mm, and 8 mm, respectively. Using these values, the simulated radiation pattern is shown in Fig. 6b by a solid line. To have a Huygens source, an electric and a magnetic dipole perpendicular to each other is required. In order to show how electric and magnetic dipoles are created, the current distribution on the unit cell is plotted in Fig. 6a. As is seen in this figure, the magnetic dipole is generated due to the rotation of electric current. Now, for the unit cell of Fig. 5a, the length of *L*1 is considered equal to the optimal value of *L*, and the Length of *L*2 is selected as the phase difference between elements *L*1 and *L*2 is equal to $180{^{\circ }}$ to eliminate specular reflection. For this purpose, the phase variation of the reflected wave for different lengths of *L* is obtained using the CST simulator (by the assumption of infinite periodicity for sub-unit cell) as shown in Fig. 6c. According to the curve of this figure, the required phase difference is obtained for $L2 = 5$ mm, therefore the design of the unit cell is completed. Using these calculated lengths, in Fig. 7a, the radiation pattern is plotted for an array with $21 \times 21$ unit cells. As can be seen, the specular reflection is not completely removed in this case. Actually, when the dipoles are placed together in an array, due to the differing mutual coupling between the dipoles with the length of *L*1 and *L*2 (with those assumed in the CST periodic boundary condition), a slight error occurs in the adjusted 180-degree phase difference^43^. Therefore, it is necessary to make a slight change in length of *L*2 to compensate for the explained error and obtain the required 180-degree phase difference to remove the specular reflection wave. The new value of *L*2 would be equal to 4.8 mm. The radiation pattern for the structure designed with these new values is shown in Fig. 7b, in which only the desired mode is propagated and other modes are eliminated.

## Design of a QHGS in a realistic problem

**Figure 8:**
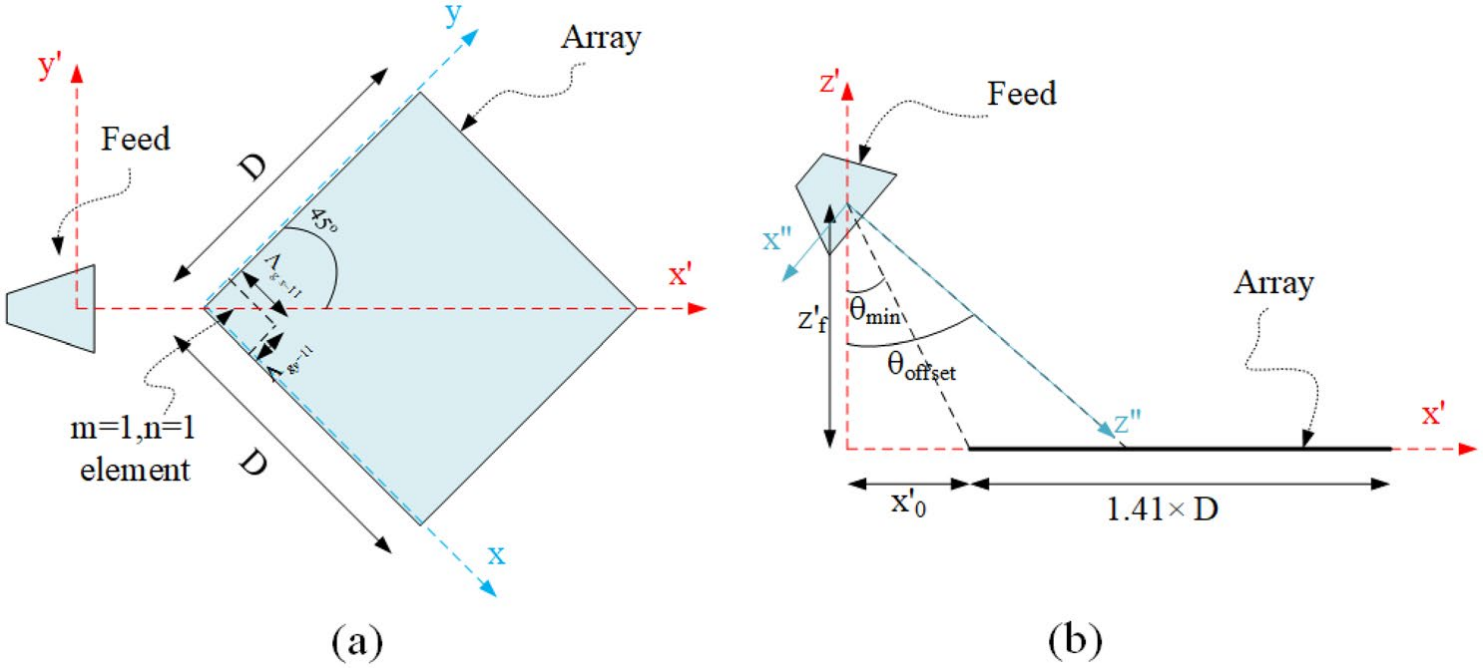
(**a**) Top view and (**b**) side view of the designed structure schematic.

In this section, the 2D grating surface designed in the previous section is employed in a real problem that is illuminated by a real feed such as a horn antenna. This surface is placed in the far field distance of the horn antenna. Therefore, the ray tracing method is employed to approximate the radiation waves from this feed. Since the illuminated wave, in this case, have a spherical wavefront, they reach each of the unit cells (including quaternary Huygens elements) with different angles, as shown in Fig. 8. As a result, it is necessary to change the period of these unit cells locally according to relation (Eq. 4) to collimate the reflected waves of them and give a directional radiation pattern.

Without loss in generality, the design is done to have the desired beam in the direction of $\left( {{{70}^{\circ }},{{225}^{\circ }}} \right)$. To use quaternary Huygens elements in the design of the desired grating surface, the feed should be located as the waves impinge all of the elements with an appropriate angle in which 3 or 4 propagating modes exist. For this purpose, the arrangement of the structure and the position of the feed are considered according to Fig. 8. In this figure, if ${{\theta _{\min }}}$ is equal to $20^\circ$ (see Fig. 3), all the elements are illuminated by the appropriate angles. Two parameters of ${x'_0}$ and ${z'_f}$ are unknown in Fig. 8b and they are related to each other as Eq. (9). These parameters are determined from the ${z'_f}/D$ ratio to have the maximum achievable aperture efficiency.

9 $$\begin{aligned} {z'_f} = \frac{{{{x'}_0}}}{{\tan {\theta _{\min }}}} \end{aligned}$$

**Figure 9:**
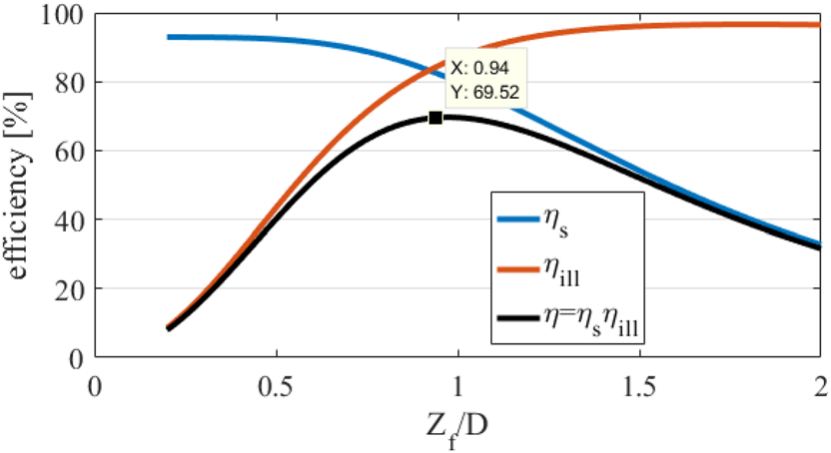
Spillover and illumination efficiencies and their multiplication versus ${Z_f}^\prime /D$ ratio.

The first step of designing is to determine the dimensions of the array and the ${z'_f}/D$ ratio according to the required gain value and the maximum efficiency. According to the relationships obtained in Refs.^46,47^ and the approximated pattern of the horn antenna with $\cos ^{16} {\theta _{f}}$, the spillover efficiency, the illumination efficiency, and their multiplication in terms of the $z'_f /D$ ratio are plotted in Fig. 9, for ${\theta _{offset}} = {45^{\circ }}$. As can be seen, the optimal value of $z'_f /D$ to have the maximum efficiency is equal to 0.94. For this value of $z'_f /D$, the resulting efficiency found by the multiplication of the spillover and illumination efficiencies is equal to $69.5\%$. Assuming to have the maximum achievable efficiency, the physical dimensions of the array are obtained to realize the desired gain using the following relation^36,48^:

10 $$\begin{aligned} {10^{G\_dB/10}} = \eta \frac{{4\pi }}{{{\lambda ^2}}}{A_{projected}}\,\,\,\,\,where\,\,\,\,{A_{projected}} = \cos {\theta _0} \times D^2 \end{aligned}$$

where $\eta$ is the efficiency of the array. In the calculation of $\eta$, the projected aperture (${A_{projected}}\,$) is considered^48^. In the projected aperture, the $cos(\theta _0)$ is multiplied in directivity. Therefore, by increasing the $\theta _0$, the directivity is decreased by $cos(\theta _0)$. Since this reduction in efficiency occurs intrinsically and independently of the designed structure, in this article, it is not considered in efficiency computing. Although, the total aperture efficiency can be easily obtained by multiplying $cos(\theta _0)$ with $\eta$. Here, the array is designed to achieve 25.5 dB gain at 12 GHz frequency. The value of *D* is calculated using Eq. (10) for $\eta = 0.695$. Then the factor of $z'_f$ is easily determined. Now, ${x'_0}$ can be calculated from Eq. (9).

In the second step, the values of the periods and the particles used in each period should be determined. In this design, the local period assumption is used. This means that since the center-to-center distance of each unit cell changes slowly in comparison to the adjacent unit cells, it is assumed that it has a specific period locally. To calculate the dimensions of the periods, using Eq. (4) and according to the structure schematic in Fig. 8, the following nonlinear equations are obtained in terms of ${\Lambda _{gx}}$ and ${\Lambda _{gy}}$.

11 $$\begin{aligned}{} & {} \left( {\sqrt{2} {{x'}_0} + {x_{mn}}} \right) \left[ {\frac{\lambda }{{{\Lambda _{gx}}_{ - mn}}} + \sin {\theta _r}\cos {\varphi _r}} \right] = \left( {\sqrt{2} {{x'}_0} + {y_{mn}}} \right) \left[ {\frac{\lambda }{{{\Lambda _{gy}}_{ - mn}}} + \sin {\theta _r}\sin {\varphi _r}} \right] \end{aligned}$$

12 $$\begin{aligned} & \left[ {\left( {\frac{\lambda }{{\Lambda _{{gx - mn}} }} + \sin \theta _{r} \cos \varphi _{r} } \right)^{2} + \left( {\frac{\lambda }{{\Lambda _{{gy - mn}} }} + \sin \theta _{r} \sin \varphi _{r} } \right)^{2} } \right] \times \left[ {x_{0}^{{\prime 2}} + x_{{mn}}^{2} + y_{{mn}}^{2} + z_{f}^{2} + \sqrt 2 x_{0}^{\prime } \left( {x_{{mn}} + y_{{mn}} } \right)} \right] \\ & \quad = \left[ {x_{0}^{{\prime 2}} + x_{{mn}}^{2} + y_{{mn}}^{2} + \sqrt 2 x_{0}^{\prime } \left( {x_{{mn}} + y_{{mn}} } \right)} \right] \\ \end{aligned}$$

where $\Lambda _{gx}$ and $\Lambda _{gy}$ are the periods of the *mnth* unit cell in the directions of *x* and *y*, respectively. ($x_{mn}$, $y_{mn}$) specifies the center location of the mentioned unit cell in the *xyz* coordinate system. This position is initially considered as follows:

13 $$\begin{gathered} x_{{mn}} = \left\{ {\begin{array}{*{20}l} {x_{{(m - 1)n}} + (\Lambda _{{gx_ - mn}} + \Lambda _{{gx_ - \left( {m - 1} \right)n}} )/2} \hfill & {if\;m > 1} \hfill \\ {\Lambda _{{gx_ - mn}} /2} \hfill & {if\;m = 1} \hfill \\ \end{array} } \right. \hfill \\ y_{{mn}} = \left\{ {\begin{array}{*{20}l} {y_{{m(n - 1)}} + (\Lambda _{{gy_ - mn}} + \Lambda _{{gy_ - m\left( {n - 1} \right)}} )/2} \hfill & {if\;n > 1} \hfill \\ {\Lambda _{{gy_ - mn}} /2} \hfill & {if\;n = 1} \hfill \\ \end{array} } \right. \hfill \\ \end{gathered}$$

**Figure 10:**
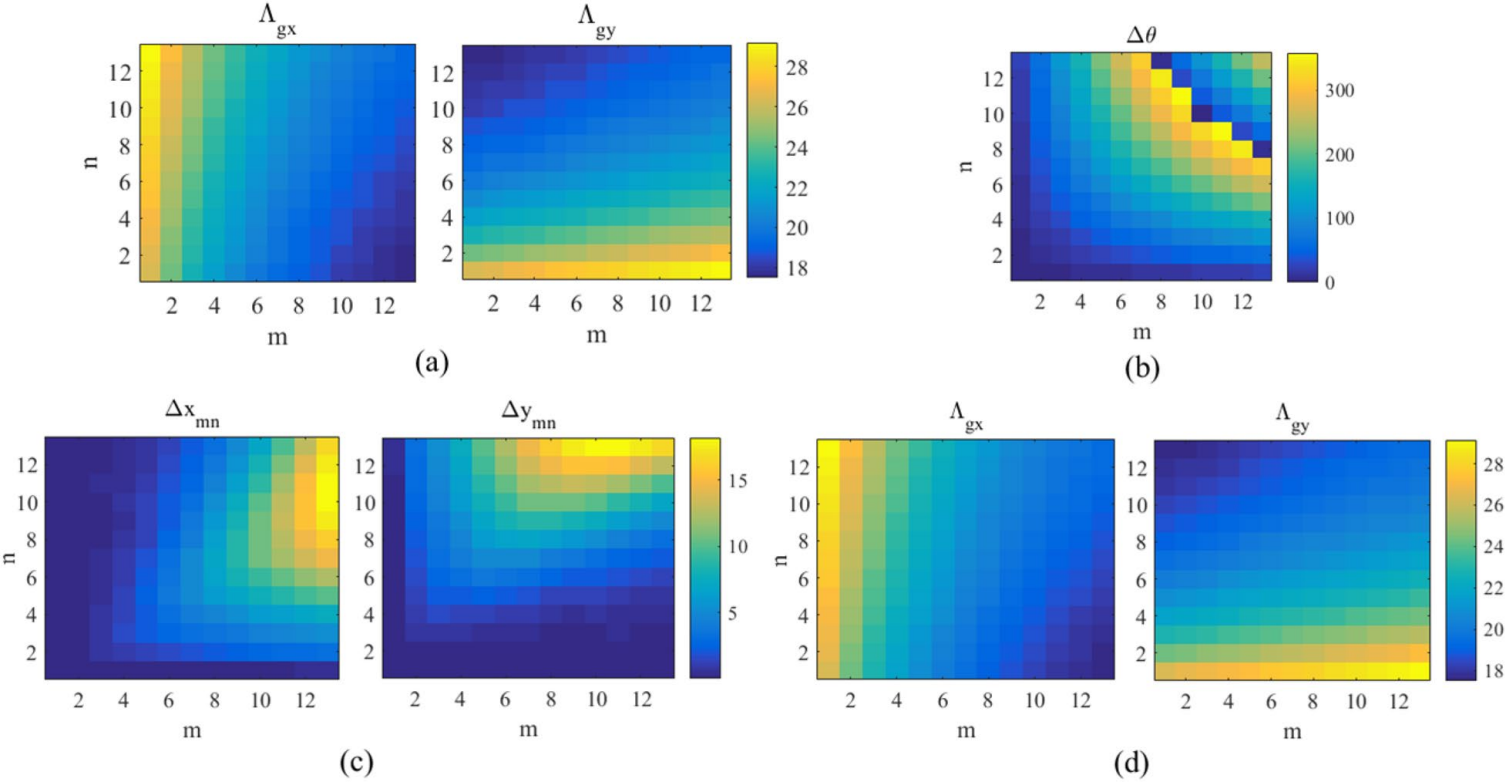
(**a**) The periods of unit cells in the *x* and *y* direction, and (**b**) the phase error caused by putting the position of the unit cell according to Eqs. (11)–(13). (**c**) The required variation in the position of the unit cells to remove phase error and (**d**) the new periods for the modified structure.

By solving this system using numerical methods, the appropriate values for ${\Lambda _{gx}}$ and ${\Lambda _{gy}}$ in each period are found. These values are shown in Fig. 10a. Now, the phase error created due to the selection of the center of periods according to Eq. (13) is checked. For this purpose, the phase difference caused by the path length difference between the first element of the (1, 1)*th* period and the first element of the other periods is calculated using the ray tracing method as follows:

14 $$\Delta \theta = k\left[ {Rd_{{mn}} - Rd_{{11}} + \left( {x^{\prime}(x_{{mn}} ,y_{{mn}} ) - x^{\prime}(x_{{11}} ,y_{{11}} )} \right)\sin \theta _{0} } \right] - 360\left\lfloor {\frac{{Rd_{{mn}} - Rd_{{11}} + \left( {x^{\prime}(x_{{mn}} ,y_{{mn}} ) - x^{\prime}(x_{{11}} ,y_{{11}} )} \right)\sin \theta _{0} }}{{360}}} \right\rfloor$$

where $Rd_{mn}$ is the distance of the 1st element in the *mnth* unit cell from the horn antenna phase center. Using Eq. (14), the generated phase error is obtained in Fig. 10b. As shown in this figure, unit cells farther from the feed cause more phase errors. To have a directional hlradiation beam, it is necessary to reduce these phase errors. For this purpose, the position of each unit cell is changed according to Eq. (15).

15 $$\begin{aligned} \left\{ {\begin{array}{*{20}{c}} {x_{mn}^{new} = {x_{mn}} - \Delta {x_{mn}},\,\,\,\,\,\,\,\,\,\Delta {x_{mn}} = \frac{{m - 1}}{{m + n - 2}}\Delta {s_{mn}}}\\ {y_{mn}^{new} = {y_{mn}} - \Delta {y_{mn}},\,\,\,\,\,\,\,\,\Delta {y_{mn}} = \frac{{n - 1}}{{m + n - 2}}\Delta {s_{mn}}} \end{array}} \right. \end{aligned}$$

**Figure 11:**
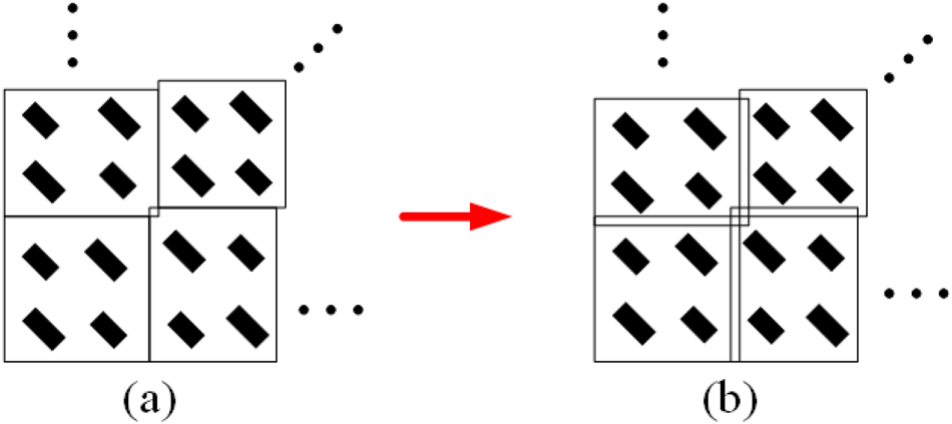
(**a**) The unit cells’ arrangement obtained according to Eqs. (13)–(15) and (**b**) the modified arrangement after removing the phase error.

**Figure 12:**
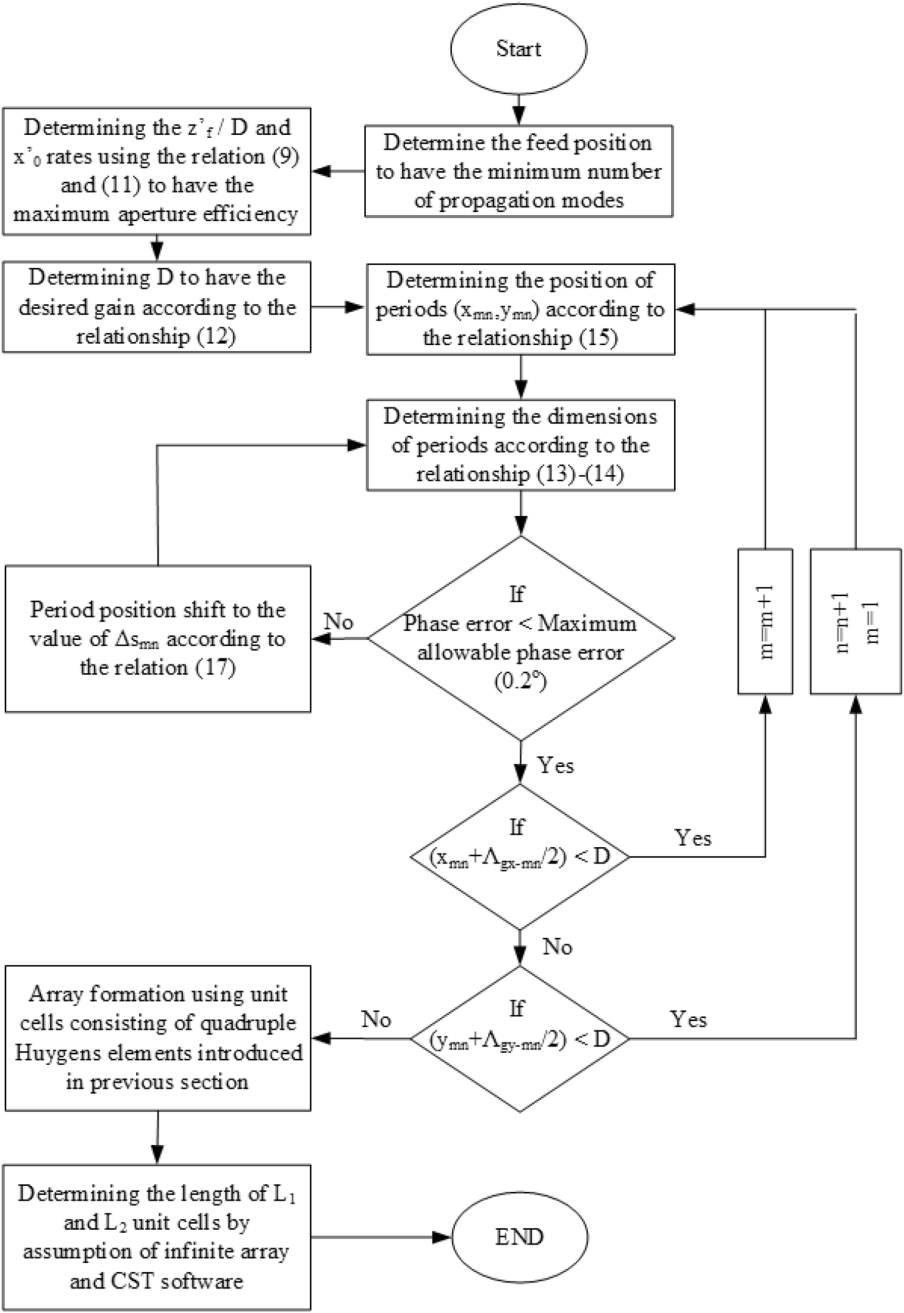
The design flowchart of a QHGS is illuminated by a real source.

In order to reduce the phase error introduced in Eq. (14) to a certain value (here $0.2^\circ$), the value of ${\Delta {s_{mn}}}$ is increased with small steps. By changing the position of the unit cells, the arrangement of the structure varies from Fig. 11a,b. As can be seen, a little interference between the unit cells is created to remove the mentioned phase error. For more clarity, the design flowchart is shown in Fig. 12. By the mentioned phase error corrections, the variations of the unit cells’ position and new periods are shown in Fig. 10c,d, respectively.

**Figure 13:**
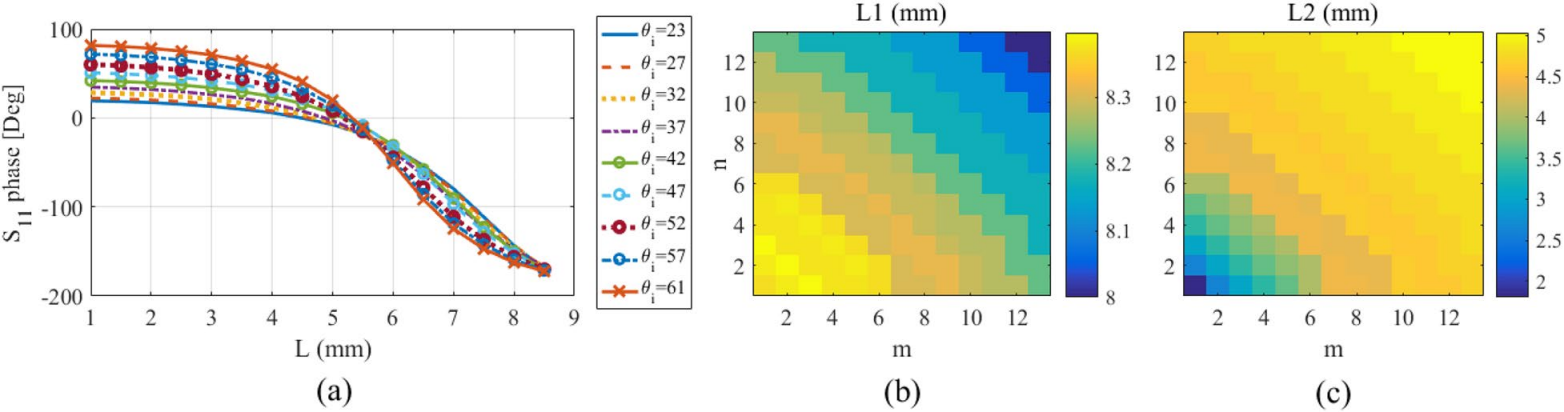
(**a**) Variations of the reflected wave phase versus dipole length, and dimensions of (**b**) *L*1 and (**c**) *L*2 extracted from these curves.

The last step is to determine the dimensions of the constituent elements. The elements in each unit cell (or each period) are the quaternary Huygens elements introduced in Fig. 5. In these elements, two parameters of *L*1 and *L*2 are unknown and their value must be determined correctly in each unit cell to minimize specular reflection. The unit cells of this designed grating surface are illuminated by an oblique incidence with elevation angles in the range of $23^\circ$–$61^\circ$. The variations of the reflected waves’ phase for different values of the incident elevation angle are drawn in Fig. 13a versus the *L* parameter with $5^{\circ }$ steps. Using these curves, *L*1 and *L*2 are found for each unit cell according to the incident angle. To create the $180^\circ$ required phase difference which is explained in the previous section, the parameters of *L*1 and *L*2 are chosen as the reflected waves of the dipoles have the phases of $-160^\circ$ and $20^\circ$ in all unit cells, respectively. The calculated values of *L*1 and *L*2 for all the unit cells are plotted in Fig. 13b,c, respectively. Now, the value of *L*2 should be optimized to compensate for the explained phase error caused by the different mutual coupling between the dipoles. After optimizing the value of *L*2, its variation range is between 4.8 and 5 mm; however, its primary value was in the range of 2–5 mm, in Fig. 13c. For simplicity, in this article, the value of $L_2$ is taken as equal to its average value which is 4.9 mm in all periods.

**Figure 14:**
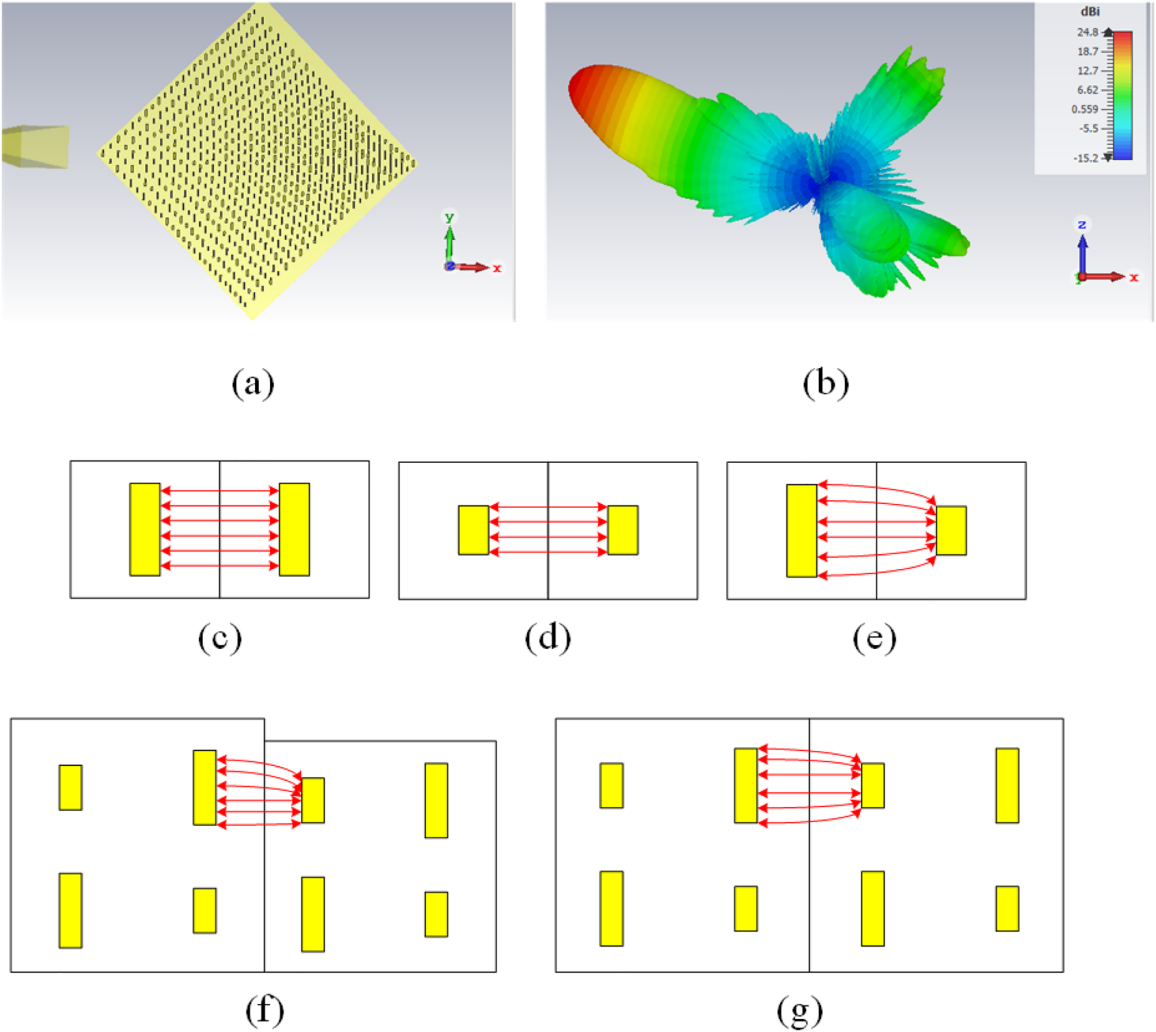
(**a**) The final structure of the designed array and (**b**) its 3D radiation pattern at the frequency of 12 GHz. (**c**)–(**e**) Coupling difference between elements inside a period by the assumption of the infinite array in CST. (**f**, **g**) Coupling difference between elements of adjacent periods by the assumption of the constant local period.

By determining the feed position, the array dimensions, the period of each unit cell, and the dimensions of the constituent elements in each period, the design procedure is completed. With these calculated values, the designed QHGS structure becomes as shown in Fig. 14a. The total designed grating surface is simulated by CST software and its 3D pattern is shown in Fig. 14b. According to this figure, the direction of the main beam is ($68^\circ$, $225^\circ$), and its SLL (side lobe level) is $-15.5$ dB. This SLL is caused due to specular reflection. Two degrees differences in the elevation angle between the simulation and the primary assumptions caused due to the limited dimensions of the array and the phase error resulting from local periodic assumption. Moreover, the realized gain is equal to 24.8 dB. This gain is equivalent to $53\%$ efficiency. In this efficiency, the projected aperture is considered.

According to the *SLL* value, it can be concluded that the mirror reflections are not completely removed, similar to the plane wave radiation in Fig. 7b. To design the unit cell and plot the $S_{11}$ phase curve in Fig. 6c of the manuscript, the infinite periodicity is assumed for each sub-unit cell in the CST simulator. Therefore, the mutual coupling between the sub-unit cells will be as shown in Fig. 14c,d for larger and smaller dipoles, respectively. However, in the designed antenna array the arrangement of the elements is as shown in Fig. 14e which results in the variation of the mutual coupling between elements. Consequently, some deviations of calculated $180^\circ$ phase differences are created in the antenna array and to remove these deviations, it is required to optimize the length of the dipoles to compensate for these errors. On the other hand, if the incident wave is a plane wave, the unit cell sizes are constant for all of the unit cells and the $S_{11}$ phase curve in Fig. 6c can be used for all of the unit cells. The lengths of the dipoles ($L_1$ and $L_2$) are the same in all of the unit cells; therefore, it is enough to optimize the $L_2$ parameter, to compensate for the mentioned error (According to Fig. 7). While for a non-plane wave incidence, the designed array contains different sizes of unit cells; consequently, there are different $S_{11}$ phase curves for each unit cells according to Fig. 13a which results in different values of $L_1$ and $L_2$ for each unit cell. Therefore, in addition to the coupling differences between the elements of a unit cell (shown in Fig. 14c–e), there are some coupling differences between the elements of neighboring unit cells. To have a better understanding, this contribution is shown in Fig. 14f,g. To compensate for these coupling differences, it is required to optimize the lengths of the dipoles in all of the unit cells simultaneously, which is a difficult and time-consuming task. To reduce this effect in our article, it is assumed that each unit-cell period is locally constant since the unit-cell period variations are slow. With this assumption, each unit cell is put in an array with a constant period equal to the period of that unit cell. Then each unit cell is irradiated by a plane wave at an appropriate angle (according to the incident angle to that unit cell in the final antenna array). In this case, the mirror reflection is reduced by optimizing the $L_2$ parameter. Although with this method, the mirror reflection of the final array is reduced, due to the error caused by the constant local period approximation, it cannot be completely removed.

**Figure 15:**
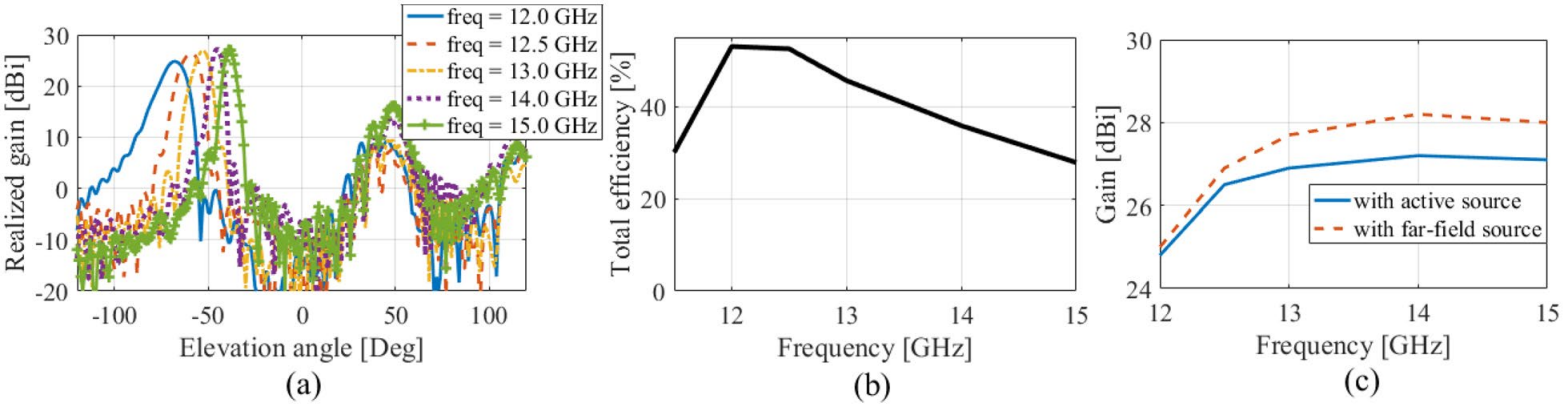
(**a**) Radiation patterns for different frequencies. (**b**) The total efficiency of the designed array versus frequency. (**c**) The effect of blockage of the feed on the antenna gain.

One of the interesting features of this designed QHGS is its capability of the beam squint, which was not possible by common reflectarrays. To show this issue, the array patterns for different frequencies are drawn in Fig. 15a. As it is seen from this figure, the direction of the main beam varies in the range of $68^\circ$–$39^\circ$ by changing the frequency from 12 to 15 GHz. The total efficiency of the array by considering the projected aperture is drawn in Fig. 15b which is about $53\%$ and its value decreases by increasing the frequency.

In the simulations done using CST software, an active horn is used to consider its blocking effect (according to Ref.^49^). But in order to better investigate this effect, the simulation with a far-field source is also done and the gains of the antenna in both states (with active horn and far-field source) are compared with each other. As shown in Fig. 15c, at the design frequency of 12 GHz, the blocking effect of the horn is insignificant because the main beam direction has not interfered with the position of the horn antenna. But when the offset angle of the main beam decreases by increasing frequency, this blockage effect increases as shown in this figure. Of course, this effect can be reduced with a little change in the position of the horn antenna in the azimuth direction to remove the interference and repeat the design based on it.

**Figure 16:**
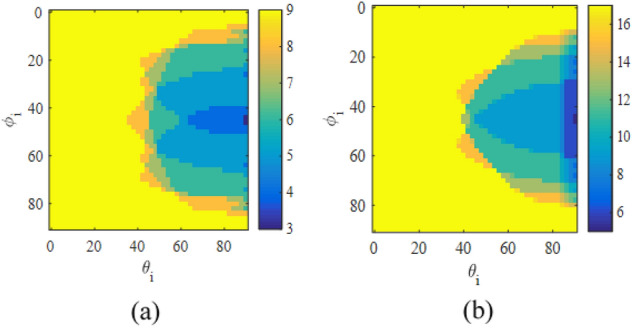
The number of propagating modes for different radiation angles and desired beams in (**a**) $15^\circ$ and (**b**) broadside.

Note that in this designed structure, due to the selection of high-order Floquet modes as a reflective mode in the desired direction, a large angle for the reflected wave is required or the angle of the incident wave to the elements should be increased. The reason for choosing high-order Floquet modes as the desired reflection mode is the limitation of the introduced unit cell to control the number of propagated modes (maximum 4 modes). Although the introduced unit cell limits the design of the array to have a beam in the broadside direction, this method can generally be used for angles close to the broadside if a suitable unit cell is designed. In this case, the desired unit cell should have the ability to control more propagated modes. For example, for the beam angle in the direction of $15^\circ$ and broadside, the minimum numbers of required propagating modes are 8 and 16, respectively; therefore, the designed unit cell should have 8 and 16 degrees of freedom to control these propagating modes, respectively. Fig. 16a,b show these issues.

## Method

The overall design process is according to the flowchart shown in Fig. 12. To determine the size and position of the periods, a MATLAB code is written for the loops shown in this flowchart. To solve the system of nonlinear equations of (11) and (12), fsolve command and function of root2d have been used in MATLAB. The unit cell boundary conditions in CST software are used to draw $S_{11}$ phase variation curves in terms of *L* length in Fig. 12a. In this simulation, the phase reference is considered exactly on the surface of the unit cell. Finally, the final structure has been simulated using CST software and the time domain solver.

## Discussion

In this article, a new method for designing anomalous reflection of radiating plane waves using two-dimensional grating surfaces based on Floquet’s theory and Huygens’ sources is presented. In these surfaces, each unit cell consists of 4 elements that have Huygens properties. The dimensions of these elements are adjusted to maximize the reflective power in the desired direction. This method is extended to the case excited by a real source, and based on it, a high-efficiency RA is designed to have the main beam in the direction of ($68^\circ$, $225^\circ$), at the frequency of 12 GHz. Our designed RA has unit cells with variable dimensions or periods. Our designed RA has beam squint capability. Although this designed array has a spatial feed similar to RAs, its performance is more similar to 2D leaky wave antennas^50–54^. As it is mentioned in Refs.^55,56^, one of the inherent drawbacks of leaky wave antennas is their low aperture efficiency (less than $30\%$) due to the exponential tapering of the aperture. But in our designed RA, it is possible to achieve higher values of the aperture efficiency (more than $53\%$), and therefore the overall efficiency and realized gain are increased at the cost of increasing the antenna profile. Table 1 shows a comparison between our work and a number of references.

**Table 1:** Comparison of the results obtained in this work with the simulated results of the other references. For all references, efficiency has been calculated using the $\eta = \frac{{{\lambda ^2}}}{{4\pi {A_p}}}\sum \nolimits _{i = 1}^N {{G_i}/\cos {\theta _{0i}}}$^31^. The $(1/\cos {\theta _0})$ factor is added in this formula due to the consideration of the projected aperture^48^. Note that NR means Not Reported.

Refs.	Operating frequency (GHz)	Aperture dimension (cm${^2}$)	Gain (dBi)	Efficiency ($\%$)	SLL	Scaning range	Beam numbers
Ref.^50^	14	$28 \times 28$	17.44	2.5	$-22$ dB	–	$N=1$
Ref.^51^	21.2	$15 \times 15$	23.5	15.9	NR	–	$N=1$
Ref.^52^	17	$24 \times 24$	18.4, 18.4	6.87	NR	$24^\circ$–$40^\circ$ $14^\circ$–$38^\circ$	$N=2$
Ref.^53^	13.5	$\pi {\left( {12.42} \right) ^2}$	24.7, 20.9	33.9	$-22.5$ dB	–	$N=2$
Ref.^41^	300	$11.025 \times 11.025$	27.57	43	NR	$-35^\circ$ to $-5^\circ$	$N=1$
This work	12	$27.5 \times 27.5$	24.8	53	$-15.5$ dB	$39^\circ$–$68^\circ$	$N=1$

## Data Availability

The datasets used and/or analyzed during the current study are available from the corresponding author on reasonable request.
